# Thomas W. Hood

**Published:** 1889-12

**Authors:** 


HENRY R. WAMPOLE & CO., 418 Arch st., Philadelphia, Pa., have been kindly handed the following beautiful samples by the gentlemanly agent of the above excellent House, to-wit: Wampole's Compound Hypophosphites; Wampole's tasteless preparation Cod Liver Oil; Wampole's concentrated extract Malt; Wampole's Syrup Hydriodic Acid; Wampole's Bromo Pyrine; Glycerine Suppositories.

